# Tracing the Zoonotic Origins of a Rare Human G5P[6] Rotavirus in Brazil

**DOI:** 10.3390/pathogens14111172

**Published:** 2025-11-17

**Authors:** Lais Sampaio de Azevedo, Vanessa Cristina Martins Silva, Yasmin França, Raquel Guiducci, Adriana Luchs

**Affiliations:** 1 Virology Center, Adolfo Lutz Institute, Av. Dr Arnaldo, nº 355, São Paulo 01246-902, SP, Brazil; 2 Graduate Program in Sciences of the Center for Disease Control, São Paulo State Department of Health, São Paulo 05403-000, SP, Brazil

**Keywords:** genetic diversity, interspecies transmission, rotavirus, gastroenteritis, molecular epidemiology

## Abstract

The porcine origin rotavirus A (RVA) G5 genotype is notable for its unique and sustained human circulation in Brazil, primarily as G5P[8] during the 1980s–2000s. This study aimed to characterize and investigate the full genome of a rare G5P[6] strain detected in 2013 (RVA/Human-wt/BRA/IAL-R406/2013/G5P[6]) to elucidate its evolutionary origin throughout RT-PCR, sequencing, and phylogenetic analysis. Whole-genome assessment revealed an atypical G5-P[6]-I1-R1-C1-M1-A8-N1-T7-E1-H1 constellation. The IAL-R406 VP7 (classified in Lineage I) was closely related to G5 strains that have circulated in both humans and pigs in Brazil for nearly three decades, showing no evidence of recent variant introduction. The VP4 P[6] (assigned as Lineage I) was genetically similar to Paraguayan and Argentinian G4P[6] porcine-like strains, indicating a regional swine reservoir and zoonotic RVA spillover in South America. The remaining nine segments support the animal–human reassortant origin of IAL-R406, showing broad similarity to porcine-like human and porcine strains described worldwide, with additional relationships to bovine (Republic of Korea, USA), feline-like human (Brazil), equine (UK), simian (Caribbean), wild boar/fox (Croatia), and classical human (Japan, USA) strains. In particular, the NSP1-A8 and NSP3-T7 genotypes, extremely rare in humans yet widespread in animals, especially swine, strongly indicate interspecies reassortment, likely resulting from porcine-to-human transmission. Together, these findings reinforce swine as a persistent reservoir for zoonotic RVA infections and highlight the importance of a One Health approach integrating human and animal surveillance to better understand RVA cross-species transmission and evolution.

## 1. Introduction

Rotavirus A (RVA), taxonomically designated as *Rotavirus alphagastroenteritidis* [1], is a leading etiological agent of acute gastroenteritis in infants and young children worldwide. In addition to its global impact on human health, RVA infects a broad spectrum of animal species, contributing to significant economic losses in livestock industries and facilitating interspecies transmission events [2,3]. As of the end of 2024, 131 countries have incorporated rotavirus vaccines into their National Immunization Programs (NIPs), reflecting a concerted global effort to mitigate the disease burden associated with RVA infection [4]. The introduction of the Rotarix^®^ vaccine in Brazil in 2006 marked a pivotal moment in pediatric public health. Between 2006 and 2018, hospitalization rates due to diarrhea among children under five years of age decreased by 52.5%, from 68.4 to 32.5 cases per 10,000 children. Moreover, diarrhea-associated mortality declined by an average of 9.8% annually, with even more pronounced reductions observed in the Northeast region (up to 13.9% per year) [5].

RVA belongs to the genus *Rotavirus*, within the family *Reoviridae*, order *Reovirales*, subfamily *Sedoreovirinae*, and the realm *Riboviria* [6]. The viral genome comprises 11 segments of double-stranded RNA (dsRNA), which encode six structural proteins (VP1–VP4, VP6, and VP7) and five to six non-structural proteins (NSP1–NSP5/6) [7]. Historically, RVA strains have been classified using a binary system based on the outer capsid proteins VP7 (G genotype) and VP4 (P genotype) [8]. The most prevalent human genotypes include G1P[8], G2P[4], G3P[8], G4P[8], G9P[8], and G12P[8] [9]. Several other genotypes, such as G5, G6, G8, G10, G11, and P[1], P[5], P[7], P[9], and P[14], have been sporadically detected in humans and are believed to result from zoonotic transmissions, highlighting the potential for interspecies reassortment [10,11,12,13].

To enhance the molecular epidemiological resolution of RVA strains, a full-genome classification system was developed, assigning genotypes to each of the 11 genome segments using the format Gx–P[x]–Ix–Rx–Cx–Mx–Ax–Nx–Tx–Ex–Hx [14]. Most human RVA strains fall into one of three major genomic constellations: Wa-like (Genogroup 1): Gx–P[x]–I1–R1–C1–M1–A1–N1–T1–E1–H1, DS-1-like (Genogroup 2): Gx–P[x]–I2–R2–C2–M2–A2–N2–T2–E2–H2, and AU-1-like (Genogroup 3): Gx–P[x]–I3–R3–C3–M3–A3–N3–T3–E3–H3 [14]. Whole-genome analyses have revealed that RVA genetic diversity arises through a combination of point mutations, gene segment reassortment (both inter- and intraspecies), and recombination [8,15]. Rare G/P-type combinations in humans often feature atypical genomic profiles, indicating possible reassortment or zoonotic origins. Representative strains include the Thai G9-P[23]-I5-R1-C1-M1-A8-N1-T1-E1-H1 [16], the Belgian G9-P[6]-I5-R1-C1-M1-A8-N1-T7-E1-H1 [17], and the Brazilian G26-P[19]-I5-R1-C1-M1-A8-N1-T1-E1-H1 [18].

Brazil is internationally recognized for its robust epidemiological and molecular surveillance of RVA, particularly following the inclusion of Rotarix^®^ in the NIP in 2006 [19]. The country is considered a hotspot for RVA genetic diversity and has reported a high frequency of rare or emerging strains, including genotypes of zoonotic origin [20,21]. Among these, the G5 genotype holds particular historical and epidemiological relevance [22,23]. Typically associated with porcine RVA strains, G5 is rarely identified in humans globally [7,10,24,25,26,27,28,29]. However, Brazil was the first, and remains one of the few, countries to report sustained human circulation of G5 strains, particularly between the 1980s and early 2000s [22,23,30,31,32,33,34,35]. The initial detections of human G5 strains in Brazil occurred in the early 1980s, exclusively in combination with the VP4 genotype P[8] [22,30,31]. Subsequent molecular surveillance revealed that G5P[8] strains co-circulated with common human genotypes (i.e., G1P[8], G2P[4], G3P[8], and G4P[8]), and reached a regional prevalence of up to 9–10%, a level unmatched elsewhere [23,32,33,34,35]. Phylogenetic studies confirmed the porcine origin of the VP7 (G5) gene, while the VP4 (P[8]) and other genomic segments were of human origin, supporting a reassortment event between porcine and human strains [23,36,37].

In May 2013, an unusual G5P[6] RVA strain (RVA/Human-wt/BRA/IAL-R406/2013/G5P[6]) was identified in an 11-year-old male patient during routine epidemiological surveillance in Goiânia, Goiás State, in the Midwestern region of Brazil. The P[6] genotype is commonly found in porcine RVA strains [38,39] and is often associated with the zoonotic transmission of animal RVA genotypes to susceptible human populations [40]. Human infections with G5P[6] strains are exceedingly rare, with cases reported only in a few countries, including China, Vietnam, Japan, Bulgaria, and Zambia [10,24,26,27,29]. To date, complete genome constellation data are available for only three of these strains (from Japan, Bulgaria, and Zambia) [10,27,29].

The present study aimed to perform full-genotype characterization and phylogenetic analysis of the RVA/Human-wt/BRA/IAL-R406/2013/G5P[6] strain in order to elucidate its evolutionary origin and assess its genomic relationship with both human and animal RVA strains. These findings contribute to the understanding of interspecies transmission dynamics and highlight the ongoing importance of molecular surveillance in detecting and monitoring emerging or rare RVA genotypes.

## 2. Material and Methods

### 2.1. Sample

The RVA/Human-wt/BRA/IAL-R406/2013/G5P[6] strain is part of a broader study investigating the evolution of animal-origin RVA strains infecting humans. This study included the whole-genome characterization of 83 samples collected in Brazil between 2007 and 2020 (Technical-Scientific Council (CTC) of the Adolfo Lutz Institute numbers: CTC 45-G/2014 and CTC 02-N/2021.

### 2.2. Viral dsRNA Extraction and RT-PCR of the 11 Genome Segments

Double-stranded RNA (dsRNA) from RVA was extracted from 10% (*w*/*v*) fecal suspensions using the QIAamp^®^ Viral RNA Mini Kit (QIAGEN, Valencia, CA, USA), following the manufacturer’s instructions.

Reverse transcription PCR (RT-PCR) targeting all 11 gene segments was performed using in-house protocols. Primers for VP1, VP2, VP3, and VP4 were based on those described by Wang et al. [41], while primers for NSP1, NSP2, NSP3, NSP4, NSP5/6, VP6, and VP7 followed the protocols of Magagula et al. [42]. Amplification conditions were applied as described by Gouvea et al. [43]. PCR products were subsequently separated by electrophoresis on 1.5% agarose gels stained with GelRed™ (Biotium, Fremont, CA, USA), alongside a 100 bp molecular size ladder. Gels were visualized using a gel documentation system.

### 2.3. Sequencing and Genotyping

PCR amplicons were sequenced using the BigDye™ Terminator v3.1 Cycle Sequencing Kit (Applied Biosystems, Foster City, CA, USA), employing the same primers used in the amplification reactions. Sequencing was performed on an ABI 3500 Genetic Analyzer (Applied Biosystems, Foster City, CA, USA) at the Premium Network of Multi-User Equipment, Institute of Tropical Medicine, University of São Paulo (IMT/FMUSP). Chromatogram quality was assessed and manually edited using Sequencher™ v4.7 software (Gene Codes Corporation, Ann Arbor, MI, USA). Genotype assignment was conducted using the Rotavirus A Genotyping Tool v0.1 [44].

### 2.4. Sequence Alignment and Phylogenetic Analysis

Sequences obtained in this study were aligned with reference RVA sequences for the NSP1–NSP5/6 and VP1–VP4, VP6, and VP7 genes, retrieved from GenBank. Multiple sequence alignments were performed using the CLUSTALW algorithm implemented in BioEdit v7.0.5.2 (Ibis Therapeutics, Carlsbad, CA, USA). Maximum likelihood (ML) phylogenetic trees were constructed for each gene segment using MEGA X [45], with the best-fitting nucleotide substitution models determined based on the corrected Akaike Information Criterion (AICc). The following models were applied: General Time Reversible (GTR) + G + I for NSP1, VP2, and VP3; GTR + G for VP1; Tamura 3-parameter (T92) + G + I for NSP2, NSP5, VP4, VP6, and VP7; T92 + G for NSP3; and Hasegawa–Kishino–Yano (HKY) + G for NSP4. Branch support was evaluated with 1000 bootstrap replicates. Pairwise nucleotide identities were calculated using distance matrices generated in MEGA X [45].

Reference strains were included for Lineage assignment based on previously published studies. For the VP4 P[6] genotype, five distinct lineages (I–V) have been previously established by Martella et al. [46]. For the VP7 G5 genotype, three major lineages (I–III) have been described, as reported by da Silva et al. [23].

## 3. Results

Near-complete sequences were obtained for the NSP1–NSP5/6, VP1, VP2, VP6, and VP7 segments, while partial sequences were generated for the VP3 and VP4 genes of the RVA/Human-wt/BRA/IAL-R406/2013/G5P[6] strain. Whole-genome analysis classified the strain as G5-P[6]-I1-R1-C1-M1-A8-N1-T7-E1-H1, rarely reported in humans. Table 1 presents the genotype constellation of the RVA/Human-wt/BRA/IAL-R406/2013/G5P[6] strain in comparison with reference porcine and porcine-like human RVA strains.

**Figure 1:**
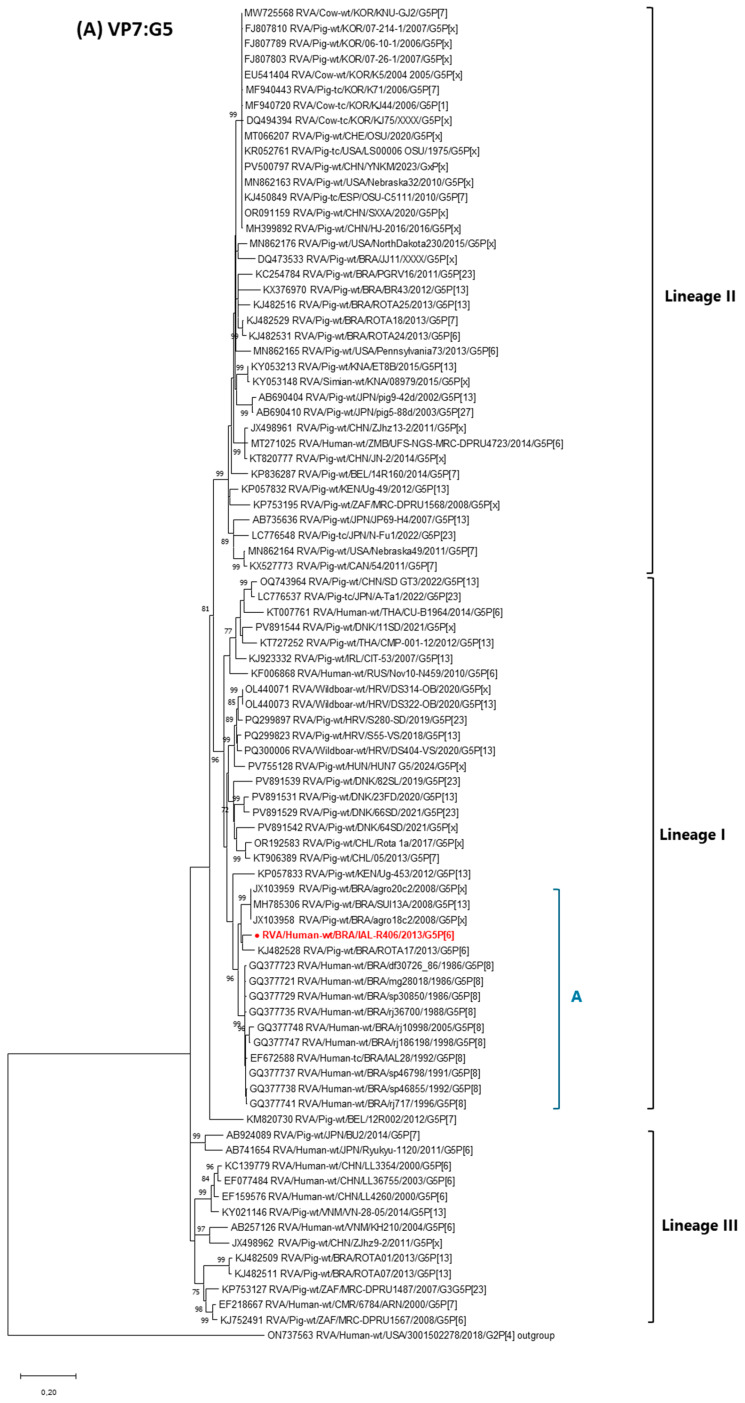

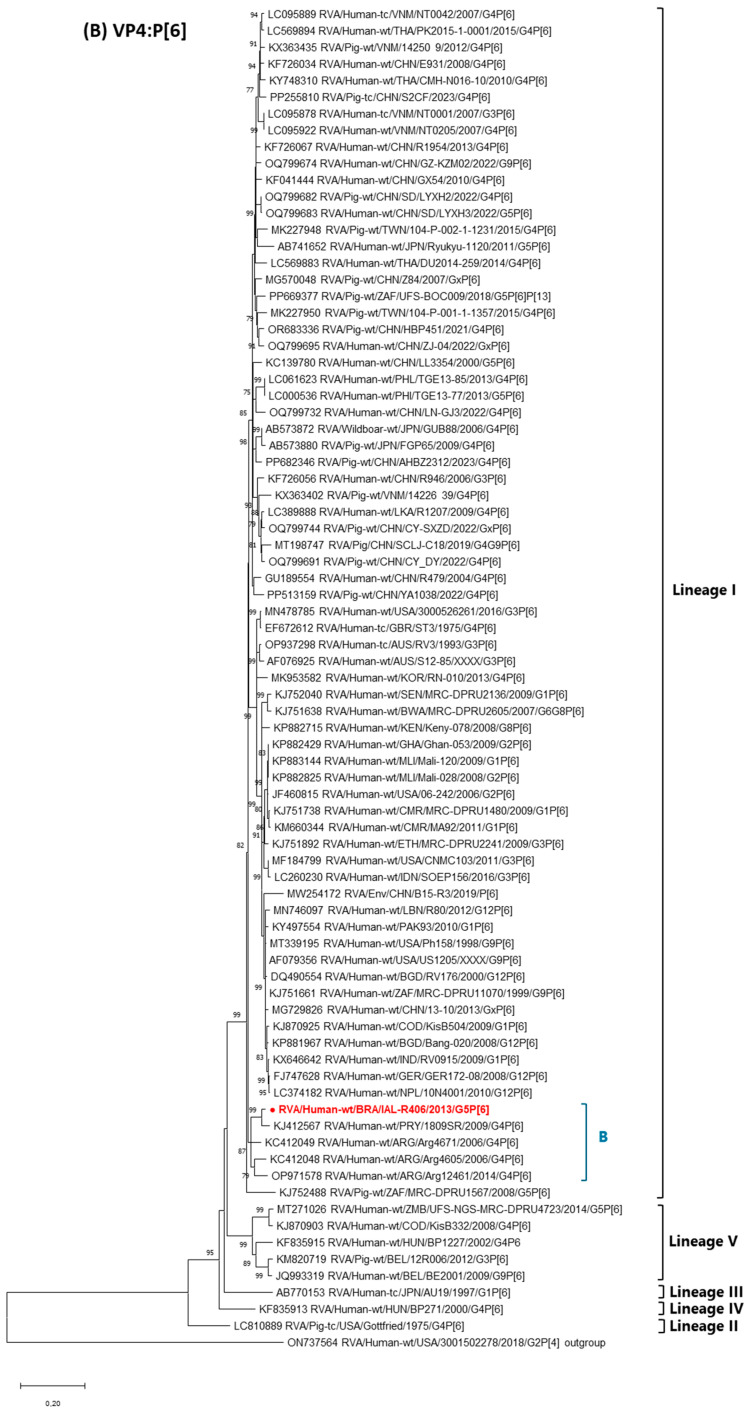

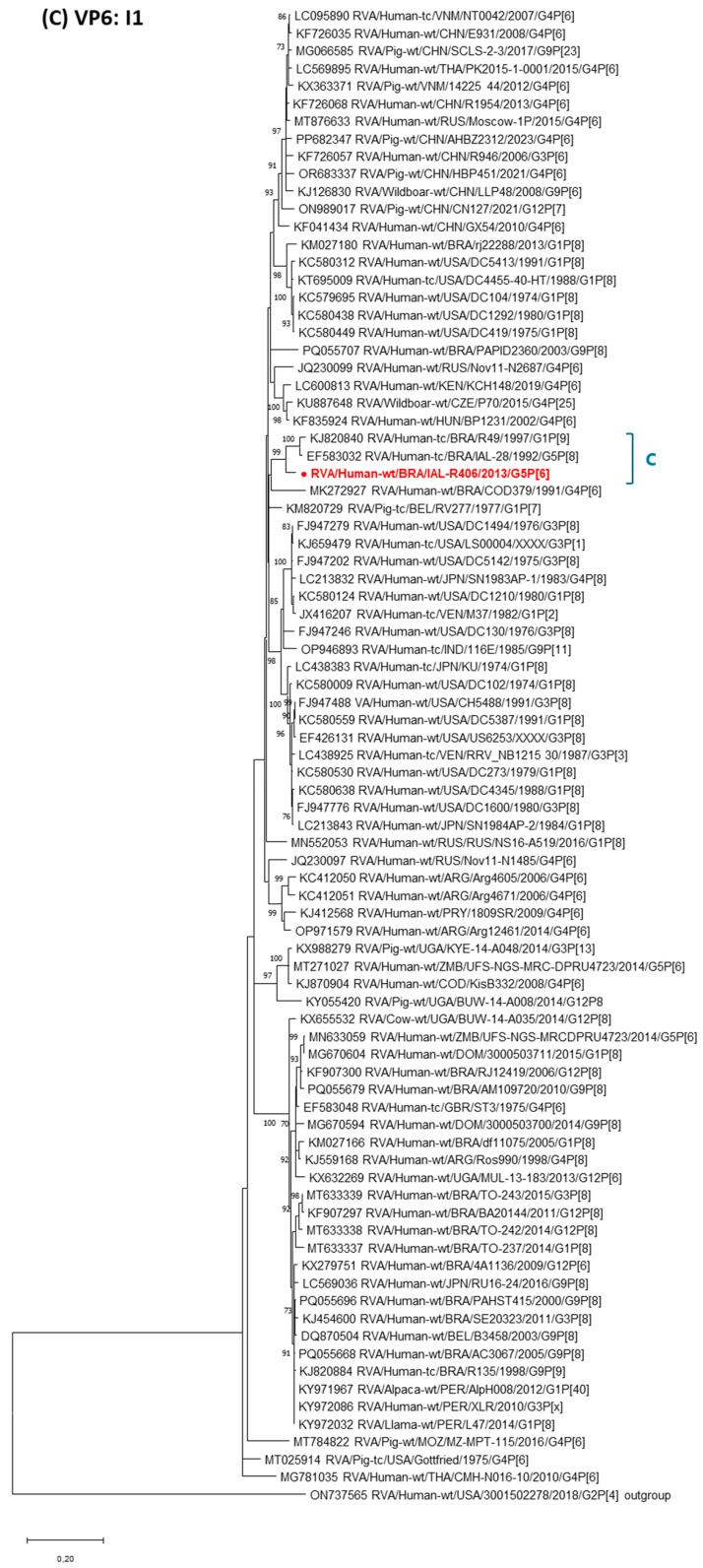

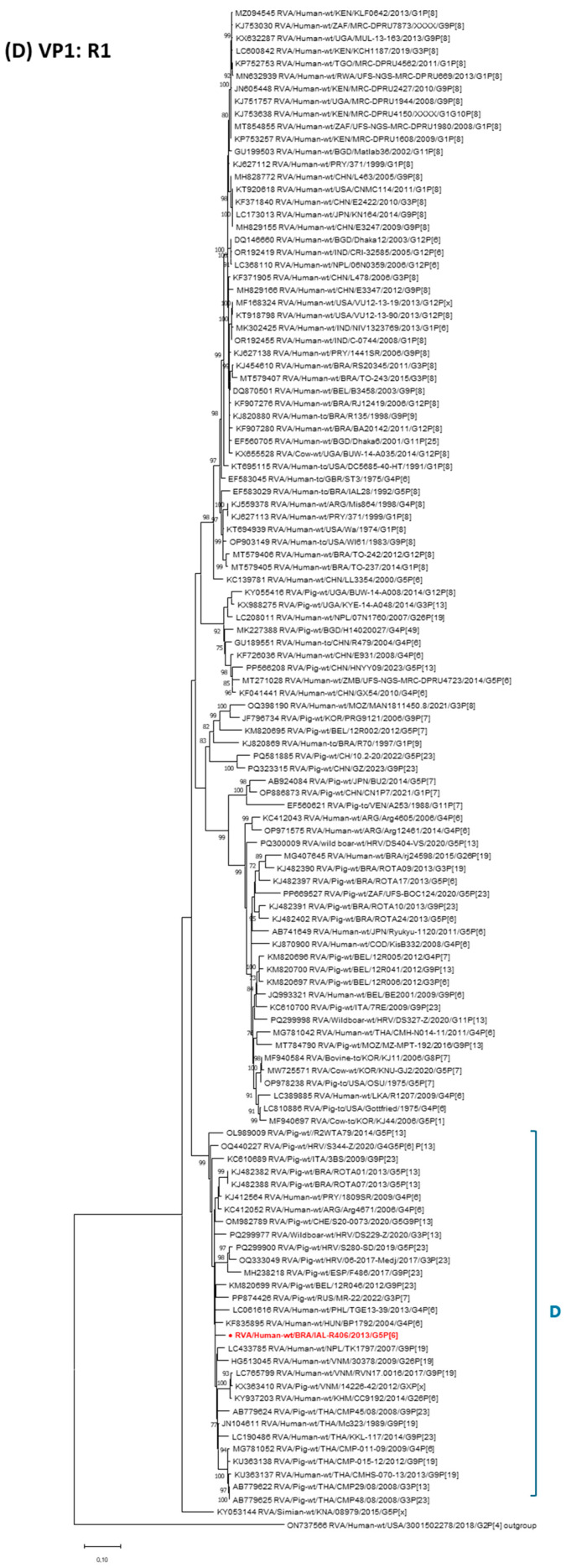

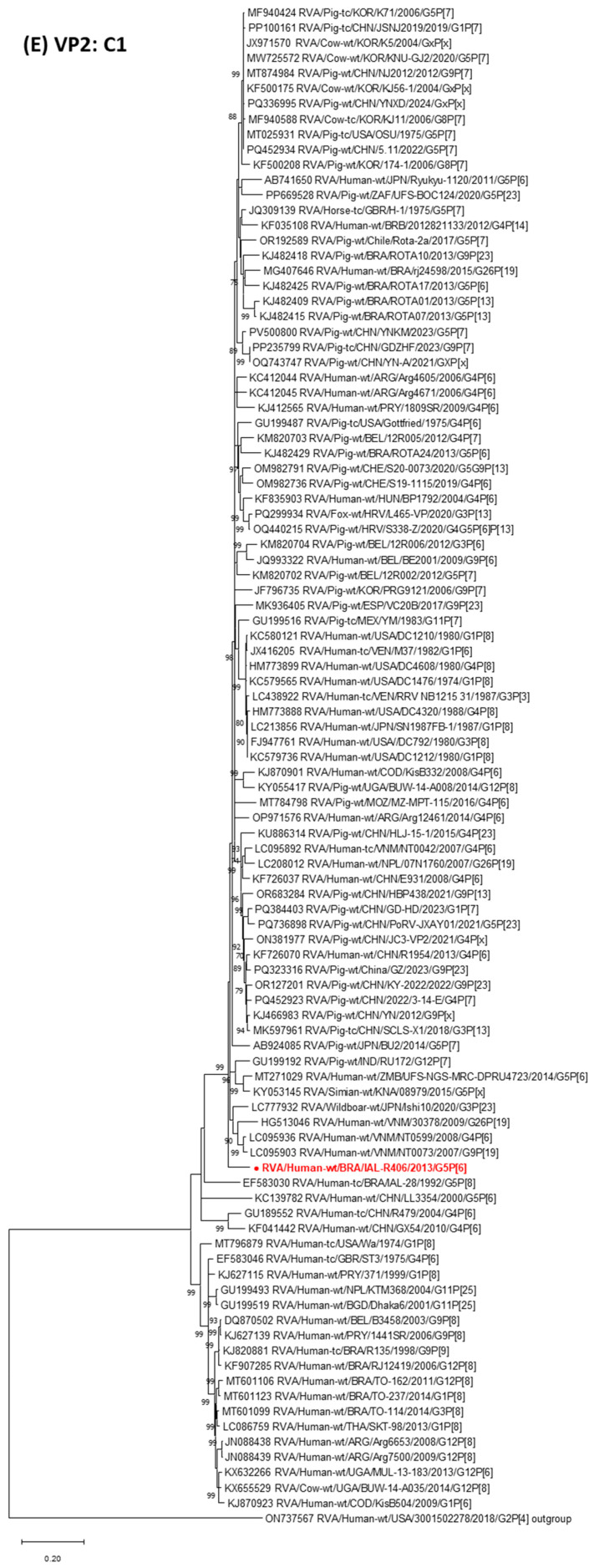

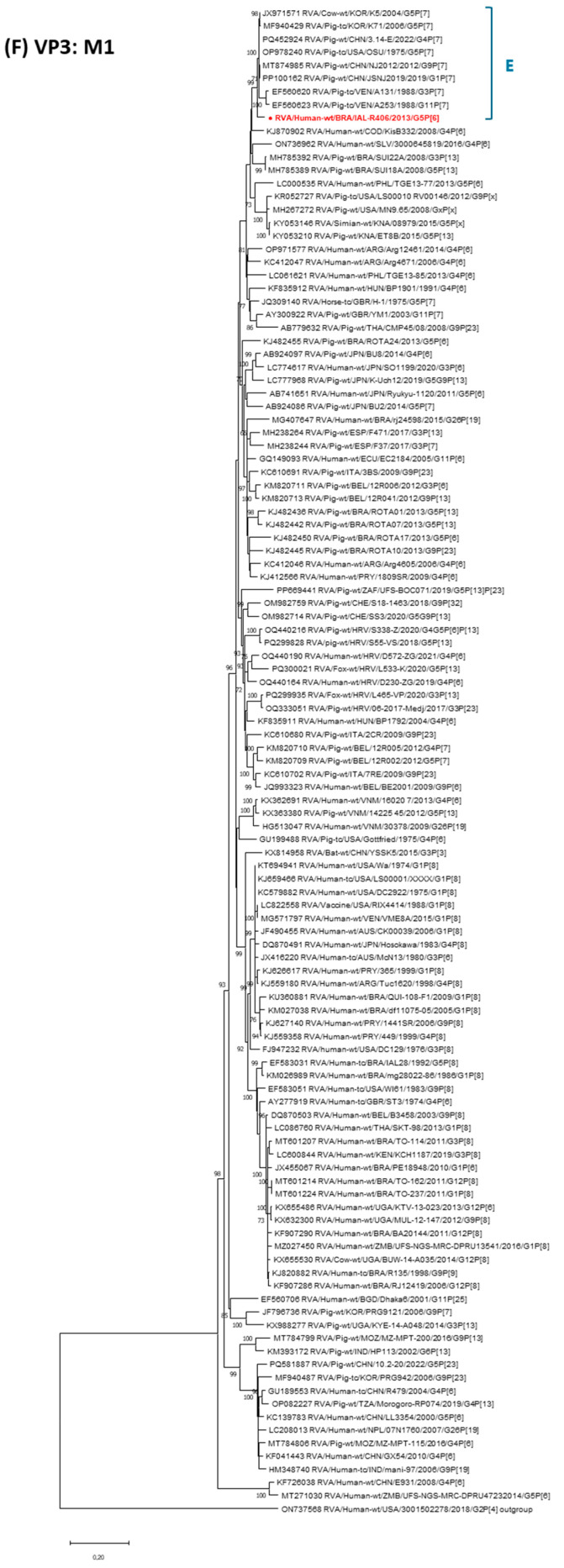

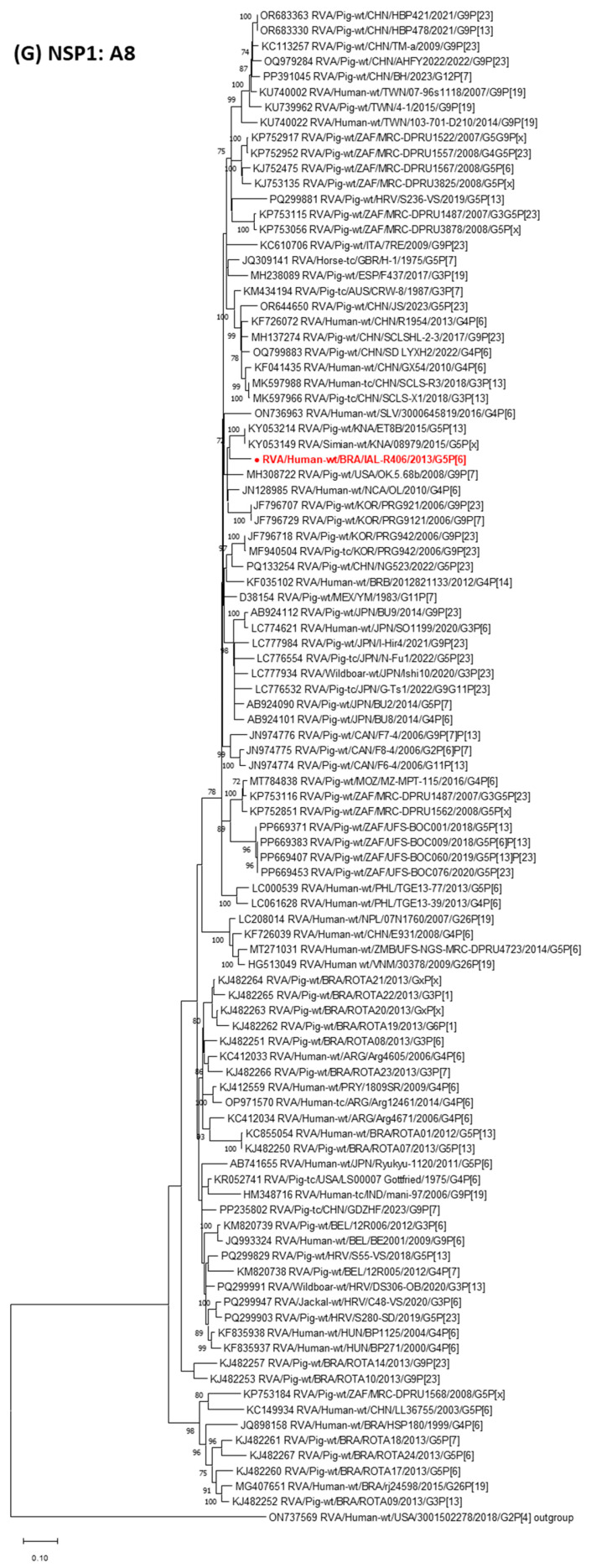

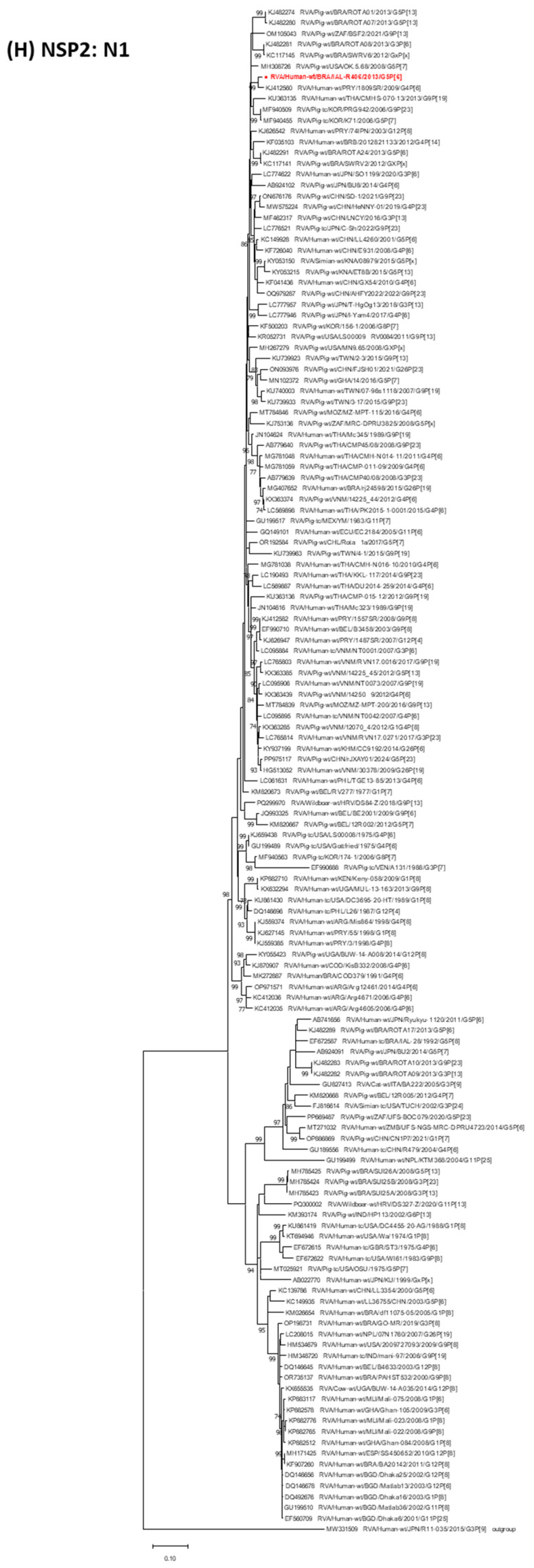

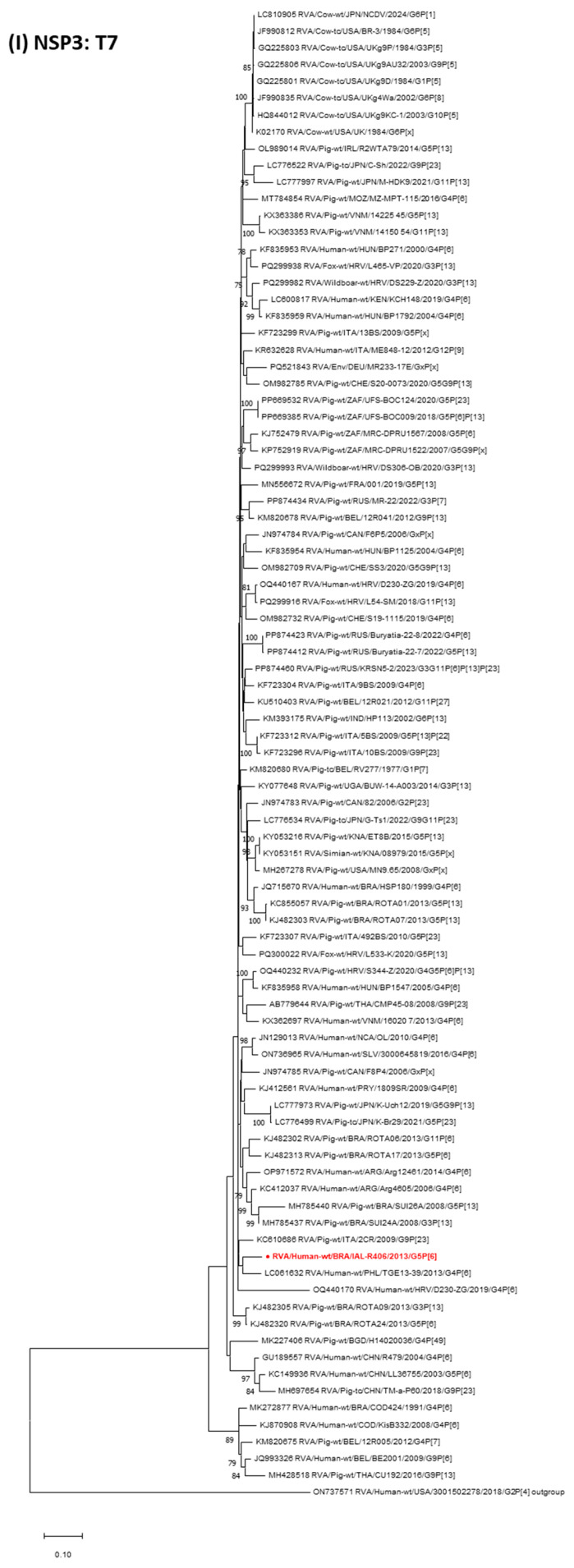

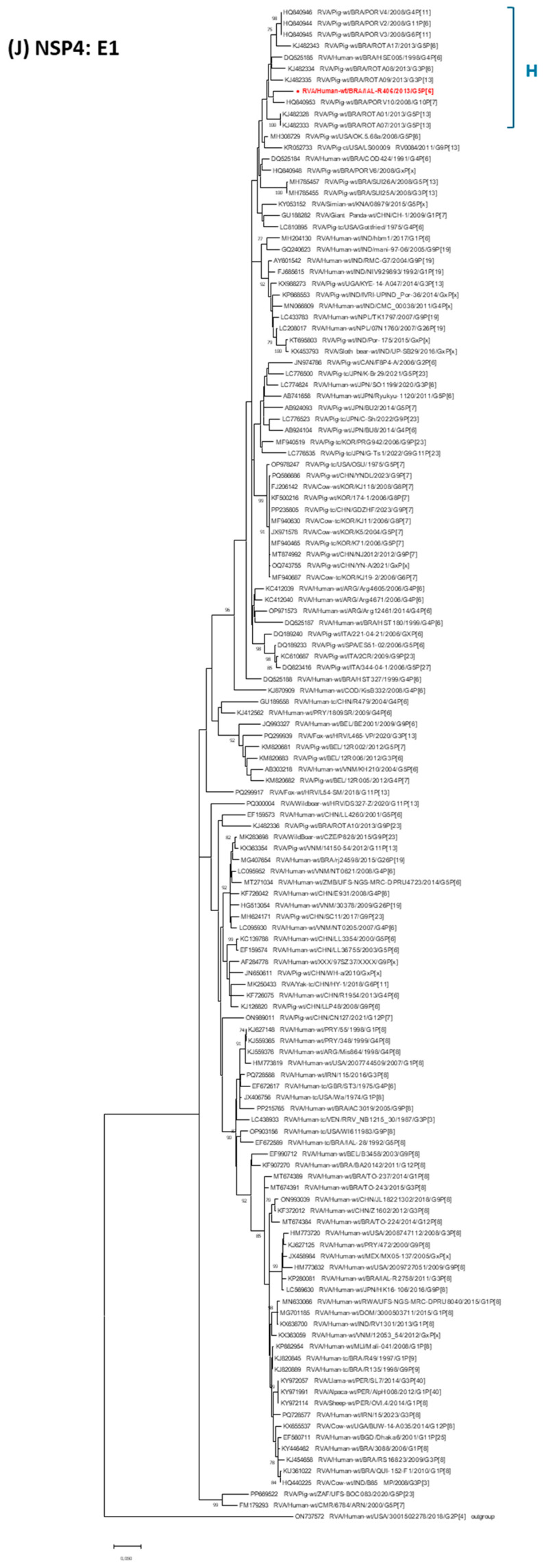

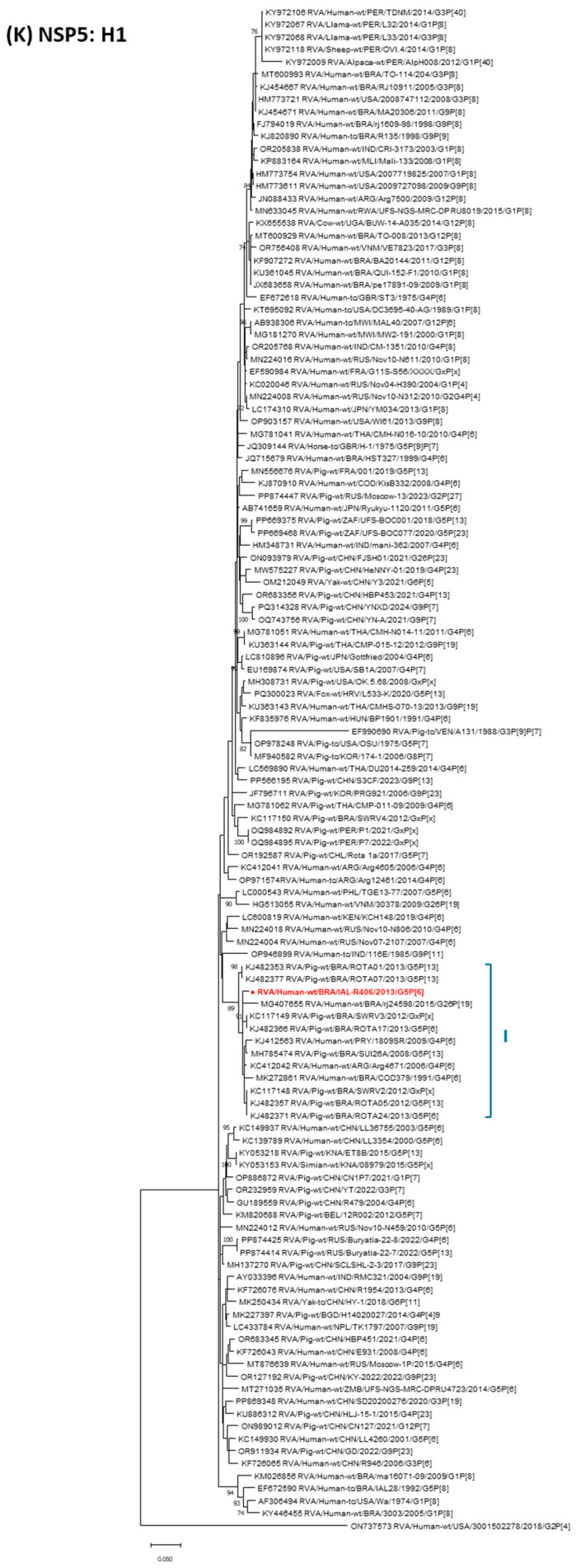
Maximum likelihood phylogenetic trees based on nucleotide sequences of 11 genome segments [VP7-G5 (A), VP4-P[6] (B), VP6-I1 (C), VP1-R1 (D), VP2-C1 (E), VP3-M1 (F), NSP1-A8 (G), NSP2-N1 (H), NSP3-T7 (I), NSP4-E1 (J) and NSP5-H1 (K)] were constructed using MEGA X software to assess the genetic relatedness of the RVA/Human-wt/BRA/IAL-R406/2013/G5P[6] strain (highlighted in bold red) to global reference strains. Bootstrap values are indicated at the nodes and scale bars represent nucleotide substitutions per site. Letters A to K denote specific subgroups identified within each gene segment.

Phylogenetic analysis of RVA G5 strains corroborated the three major monophyletic clusters previously described by da Silva et al. [23]. Strain RVA/Human-wt/BRA/IAL-R406/2013/G5P[6] (988 bp) grouped within Lineage I (bootstrap ≥ 96), which includes Brazilian human and porcine strains collected from 1986 to 2013, along with related swine (including wild boar) and human strains detected across Africa, Europe, Asia, and South America between 2007 and 2024 (Figure 1A). The VP7 gene of strain RVA/Human-wt/BRA/IAL-R406/2013/G5P[6] shared 92.2–94.5% nucleotide identity with G5 strains circulating in Brazil for nearly three decades in both humans and pigs. Among porcine strains, the closest relationships were observed with samples from the Amazon region (agro18c2/2008, agro20c2/2008, SUI13A/2008), and the states of São Paulo and Mato Grosso (ROTA17/2013, ROTA01/2013, ROTA07/2013, ROTA24/2013), encompassing the Northern and Southeastern area of the country. Among human strains, the most similar sequences came from the Midwestern and Southeastern regions, including Rio de Janeiro (rj186198_98/1998, rj36700_88/1988, rj717_96/1996, rj10998_05/2005), São Paulo (sp30850_86/1986, sp46798_91/1991, IAL-28/1992, sp46855_92/1992), Minas Gerais (mg28018_86/1986) and the Federal District (df30726_86/1986). All Brazilian RVA G5 samples clustered together into a distinct group, designated here as “A” (bootstrap ≥ 96), within Lineage I (Figure 1A).

The VP4 gene (1542 bp) was analyzed in comparison with the five established P[6] lineages (I–V) defined by Martella et al. (2006) [46]. The Brazilian RVA/Human-wt/BRA/IAL-R406/2013/G5P[6] strain fell within Lineage I (bootstrap ≥ 99), forming a subgroup with porcine-like human G4P[6] strains from Paraguay and Argentina (bootstrap ≥ 87), designated here as “B”. Brazilian RVA/Human-wt/BRA/IAL-R406/2013/G5P[6] strain was most closely related to the porcine-like human strain RVA/Human-wt/PRY/1809SR/2009/G4P[6] from Paraguay (96.8% nt, bootstrap ≥ 99), followed by the porcine-like human Argentinean RVA/Human-wt/ARG/Arg4671/2006/G4P[6] (93.7% nt). Similarities with swine strains from the same Lineage ranged from 88.9% to 93.1%. It is important to highlight that previously reported Brazilian P[6] strains of human and animal origin could not be included in the current phylogenetic analysis due to alignment incompatibilities with the available partial P[6] gene sequences in GenBank (Figure 1B).

The VP6 gene of RVA/Human-wt/BRA/IAL-R406/2013/G5P[6] strain (1294 bp) clustered (named as cluster “C”) with strong support (bootstrap ≥ 99) together with the Brazilian human prototype strains RVA/Human-tc/BRA/IAL-28/1992/G5P[8] and RVA/Human-tc/BRA/R49/1997/G1P[9], showing 94.5% and 93.9% nucleotide identity, respectively (Figure 1C).

Phylogenetic analysis based on the VP1 gene (3284 bp) showed that RVA/Human-wt/BRA/IAL-R406/2013/G5P[6] strain exhibited the closest genetic relationship to the Thai porcine-like human strain RVA/Human-wt/THA/Mc323/1989/G9P[19] (95.3% nt). It also grouped within a branch with robust support (bootstrap ≥ 99), here referred to as “D”, alongside swine, human, and porcine-like human strains detected globally, including pig-derived samples from Brazil (90.5–94.9% nt) (Figure 1D).

The RVA/Human-wt/BRA/IAL-R406/2013/G5P[6] VP2 gene (2641 bp) displayed comparable nucleotide homology (91.3–91.9% nt) with several strain sets: porcine-like human strains from Vietnam (NT0599/2008/G4P[6], NT0073/2007/G9P[19]), Venezuela (M37/1982/G1P[6]), Argentina (Arg12461/2014/G4P[6]) and Barbados (2012821133/2012/G4P[14]); classical human G1P[8]/G4P[8]/G3P[8] strains detected in the USA and Japan between 1974 and 1987 (91.1–91.7% nt); porcine strains from China, Republic of Korea and the USA identified between 1975 and 2024 (91.4–91.8% nt); bovine strains from Republic of Korea collected between 2004 and 2020 (91.3–91.7% nt); and an equine strain from UK (H-1/1975/G5P[7]) (91.4% nt) (Figure 1E).

The VP3 gene (2508 bp) of the strain RVA/Human-wt/BRA/IAL-R406/2013/G5P[6] clustered within a well-supported group (bootstrap ≥ 99, designated “E”) composed of porcine and bovine strains from Asia (Republic of Korea, China) and the Americas (USA, Venezuela). This strain showed the highest nucleotide sequence identity (97.1%) with the Korean bovine strain RVA/Cow-wt/KOR/K5/2004/G5P[7], the Chinese porcine strain RVA/Pig-wt/CHN/NJ2012/2012/G9P[7] and the porcine prototype strain RVA/Pig-tc/USA/OSU/1975/G5P[7]. It also exhibited high similarity (96.7–97.1% nt) to additional Asian porcine strains, including RVA/Pig-tc/KOR/K71/2006/G5P[7], RVA/Pig-wt/CHN/3.14-E/2022/G4P[7] and RVA/Pig-wt/CHN/JSNJ2019/2019/G1P[7]. In contrast, lower sequence identities (93.1–93.4% nt) were observed with Venezuelan porcine strains RVA/Pig-tc/VEN/A131/1988/G3P[7] and RVA/Pig-tc/VEN/A253/1988/G11P[7]. Brazilian porcine strains were more divergent, showing overall nucleotide similarities ranging from 88.4% to 94.2% when compared to the RVA/Human-wt/BRA/IAL-R406/2013/G5P[6] strain (Figure 1F).

The NSP1 gene (1434 bp) of RVA/Human-wt/BRA/IAL-R406/2013/G5P[6] strain was most similar (90.7% nt) to two animal-origin strains detected on the island of St. Kitts, Caribbean region, in 2015: the porcine strain RVA/Pig-wt/KNA/ET8B/2015/G5P[13] and the primate strain RVA/Simian-wt/KNA/08979/2015/G5P[x] (bootstrap ≥ 72, cluster “F”) (Figure 1G).

The strain RVA/Human-wt/BRA/IAL-R406/2013/G5P[6] displayed the maximum nucleotide sequence identity with the NSP2 (959 bp) nucleotide sequence of Argentinian porcine-like human RVA/Human-wt/PRY/1809SR/2009/G4P[6] at 97.8% nt (bootstrap ≥99, cluster “G”) (Figure 1H).

The strain RVA/Human-wt/BRA/IAL-R406/2013/G5P[6] belongs to the rare NSP3 T7 genotype (892 bp) and exhibited the closest genetic relationship with a variety of animal-like human strains, such as ITA/ME848-12/2012/G12P[9], HRV/D230-ZG/2019/G4P[6], PHL/TGE13-39/2013/G4P[6], KEN/KCH148/2019/G4P[6], PRY/1809SR/2009/G4P[6], HUN/BP271/2000/G4P[6], NCA/OL/2010/G4P[6], SLV/3000645819/2016/G4P[6], HUN/BP1125/2004/G4P[6] and ARG/Arg4605/2006/G4P[6], with nucleotide identities ranging from 91.1% to 92.8%. Comparable identities (91.1–92.2% nt) were observed with domestic animals strains, mainly pigs, from Russia (KRSN5-2/2023/G3G11P[6]P[13]P[23]), Canada (F8P4/2006/GxP[x]), South Africa (UFS-BOC124/2020/G5P[23], MRC-DPRU1567/2008/G5P[6], UFS-BOC009/2018/G5P[6]P[13]), Japan (C-Sh/2022/G9P[23]), India (HP113/2002/G6P[13]), Italy (2CR/2009/G9P[23], 13BS/2009/G5P[x], 9BS/2009/G4P[6], 5BS/2009/G5P[13]P[22]), Ireland (R2WTA79/2014/G5P[13]), Switzerland (S20-0073/2020/G5G9P[13], S19-1115/2019/G4P[6]) and Belgium (RV277/1977/G1P[7]). The strain also showed similarity with the USA bovine strain UK/1984/G6P[x] (91.3% nt) and with wild animal strains from Croatia, including wild boars (DS306-OB/2020/G3P[13], DS229-Z/2020/G3P[13]; 91.7–92.1% nt) and foxes (L465-VP/2020/G3P[13], L54-SM/2018/G11P[13]; 91.3–91.7% nt) (Figure 1H).

Phylogenetic analysis based on the NSP4 gene (633 bp) revealed that the Brazilian strain RVA/Human-wt/BRA/IAL-R406/2013/G5P[6] shares the highest nucleotide identity (95.6%) with the porcine strain RVA/Pig-wt/BRA/PORV10/2008/G10P[7], also detected in Brazil. The porcine origin of the NSP4 gene is further supported by its close genetic relationship with several Brazilian porcine RVA strains, including RVA/Pig-wt/BRA/PORV4/2008/G4P[11], PORV2/2008/G11P[6], PORV3/2008/G6P[11], ROTA17/2013/G5P[6], ROTA08/2013/G3P[6], ROTA09/2013/G3P[13], ROTA01/2013/G5P[13] and ROTA07/2013/G5P[13], which exhibit high nucleotide similarity to IAL-R406 strain (93.4–95.4%). Moreover, a Brazilian porcine-like human strain, RVA/Human-wt/BRA/HSE005/1998/G4P[6], previously detected in neonates and young children in Belém, displayed 94.9% nucleotide identity and clustered within the same group (here named cluster “H”) (Figure 1J), further supporting the zoonotic potential of this strain.

Finally, the RVA/Human-wt/BRA/IAL-R406/2013/G5P[6] NSP5 gene (651 bp) was nearly identical (99.2% nt) to the Argentinian porcine-like human strain RVA/Human-wt/ARG/Arg4671/2006/G4P[6]. It grouped within cluster “I” (bootstrap ≥89) together with Brazilian swine strains detected between 2008 and 2013 (98.3–99.0% nt) and porcine-like human strains identified in Brazil (rj24598/2015/G26P[19], COD379/1991/G4P[6]) and Paraguay (1809SR/2009/G4P[6]) over different decades (97.9–98.2% nt) (Figure 1K).

**Figure 2:**
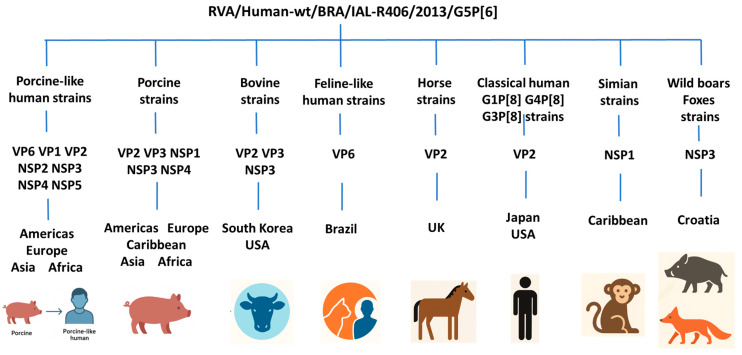
Graphical illustration of the global distribution and host origins of the 11 genomic segments of strain RVA/Human-wt/BRA/IAL-R406/2013/G5P[6].

Figure 2 shows a graphical overview of the global distribution and host origins of the 11 genomic segments of strain RVA/Human-wt/BRA/IAL-R406/2013/G5P[6], summarizing their geographic locations and host species of detection.

## 4. Discussion

The complete genome characterization of the RVA/Human-wt/BRA/IAL-R406/2013/G5P[6] strain described here enhances the understanding of the genetic diversity and evolution of RVA in both human and animal populations in Brazil. This finding is particularly relevant given the unique epidemiological history of the G5 genotype in the country compared to other regions of the world [7,10,22,23,24,26,27,29,30,31,32,33,34,35]. The association of this strain with the P[6] genotype, prevalent in swine herds [28,38,39], suggests a distinct zoonotic event, separate from the historical emergence of human G5P[8] strains in Brazil in the 1980s [23,36,37].

The relatedness between the VP7-coding gene of porcine and human RVA strains has long been reported for the G5 genotype in Brazil [23,31,37] and the data obtained in this study support this hypothesis. Previous studies suggested that G5 became established and adapted to humans in Brazil in the 1980s, with no evidence of new variant introductions up to 2005 [23]. Indeed, the phylogenetic analysis conducted revealed no recent introduction of G5 into the country, indicating that the same variant has been circulating in humans and animals for decades. Notably, the G5 genotype lost epidemiological relevance after the early 2000s, especially following Rotarix^TM^ introduction, and has not persisted in the Brazilian population, as evidenced by subsequent RVA surveillance studies [21,47,48,49,50,51,52]. The decline of G5 strains carrying P[8] coincided with the introduction of the G1P[8] live oral vaccine, suggesting that vaccine-induced selection may have reduced the fitness of P[8]-associated G5 viruses [20,50]. Similarly, G5 strains have not been consistently detected in South America region [53,54,55] or elsewhere [56,57,58,59,60,61], suggesting limited adaptability for sustained human infection. Therefore, its occasional identification in human hosts strongly indicates sporadic zoonotic transmission from a long-standing porcine reservoir. It is widely recognized that interspecies transmission and reassortment between human and animal RVAs drive RVA evolution [16,46]. Additionally, the introduction of RVA vaccines may impose selective pressure on circulating strains, potentially influencing their evolutionary dynamics and global spread, as recently observed with the emergence of equine-like G3P[8] DS-1-like strains [21,49].

The P[6] genotype is frequently linked to the emergence of novel strains in susceptible populations, enabling the successful establishment of previously uncommon G types in humans [62]. The VP4-coding gene of the Brazilian RVA/Human-wt/BRA/IAL-R406/2013/G5P[6] strain shares a close phylogenetic relationship with two South American porcine-like human strains: RVA/Human-wt/PRY/1809SR/2009/G4P[6] from Paraguay [63] and RVA/Human-wt/ARG/Arg4671/2006/G4P[6] from Argentina [64]. A close genetic relationship between Paraguayan and Argentinian porcine-like human G4P[6] strains and Brazilian swine RVA strains has also been reported [63,65]. Together, these data reinforce the hypothesis of a regional swine reservoir contributing to zoonotic transmission and cross-species spillover of RVA across South American countries. A limitation of this study was the inability to sequence the VP8*-coding region, which hindered genetic comparisons of the RVA/Human-wt/BRA/IAL-R406/2013/G5P[6] strain with other Brazilian human and animal P[6] strains and limited the competence to obtain more robust evolutionary insights. Notably, complete VP4 P[6] gene sequences from Brazilian strains are scarce, as most studies focus exclusively on partial VP8*-coding region sequences [38,52,65,66,67]. To address this limitation, future studies should consider obtaining complete VP8* sequences using next-generation sequencing approaches. In addition, prospective One Health surveillance integrating human–animal sampling would generate valuable data for direct genetic comparisons and zoonotic risk assessment. Complementary case–control studies to identify exposure factors, together with the establishment of standardized biobanks and public sequence repositories, would further facilitate phylogenetic comparisons and the evaluation of interspecies spillover events.

Considering the VP7 and VP4 genes together with the genomic backbone, RVA/Human-wt/BRA/IAL-R406/2013/G5P[6] displayed the constellation G5-P[6]-I1-R1-C1-M1-A8-N1-T7-E1-H1. This rare genomic constellation has been previously reported only once in humans, in the strain RVA/Human-wt/CHN/LL36755/2003/G5P[6] [24]. Although exhibiting the same genomic constellation, the Brazilian and Chinese strains segregated into distinct phylogenetic clusters, supporting the hypothesis of independent evolutionary pathways rather than direct intercontinental transmission. In South America, porcine-like human strains RVA/Human-wt/PRY/1809SR/2009/G4P[6], RVA/Human-wt/ARG/Arg4605/2006/G4P[6], and RVA/Human-wt/ARG/Arg12461/2014/G4P[6] also shared the same backbone constellation, differing only in the VP7 gene (G4 instead of G5) [63,64,68]. Likewise, strains from Zambia (RVA/Human-wt/ZMB/UFS-NGS-MRCDPRU4723/2014/G5P[6]), Japan (RVA/Human-wt/JPN/Ryukyu-1120/2011/G5P[6]) and Bulgaria (RVA/Human-wt/BGR/BG260/2008/G5P[6]) exhibited the same genotype profile except for NSP3 (T1 instead of T7) [10,27,29]. Additionally, the Brazilian porcine strains RVA/Pig-wt/BRA/ROTA17/2013/G5P[6] and RVA/Pig-wt/BRA/ROTA24/2013/G5P[6] displayed the same genetic constellation as the human IAL-R406/2013/G5P[6] strain, diverging solely in the VP6 genotype (I5 instead of I1) [65]. Together, these comparisons indicate that similar genomic backbones have emerged independently in different regions, reflecting both zoonotic introductions and local evolutionary dynamics.

Phylogenetic analysis of the remaining structural and non-structural genome segments of RVA/Human-wt/BRA/IAL-R406/2013/G5P[6] supported its potential animal–human reassortant origin. The strain displayed genetic relationships with classical human, porcine-like human, and feline-like human strains, as well with domestic animal strains from pigs, horses, and cattle, and wildlife strains identified in monkeys, wild boars, and foxes. Specifically, the VP6, VP1, VP2, NSP2, NSP3, NSP4, and NSP5 segments were closely related to porcine-like human strains circulating across the Americas, Europe, Asia, and Africa [16,18,39,63,64,69,70,71,72,73,74,75,76,77,78]. Additionally, the VP2, VP3, NSP1, NSP3, and NSP4 segments exhibited similarity to porcine strains from Asia, the Americas, Europe, Africa, and the Caribbean [65,70,79,80,81,82,83,84,85,86,87,88,89,90,91,92]. VP2, VP3, and NSP3 also shared genetic similarity with bovine strains from Republic of Korea and the USA [81,93,94]. Further notable relationships included VP6, which was similar to a feline-like human strain from Brazil [95]; VP2, related to a horse strain from UK [96], and classical human strains from the USA and Japan [97,98,99]; NSP1, clustering with a simian strain from St. Kitts [86]; and NSP3, closely related to strains detected in wild boars and foxes from Croatia [100,101]. The combination of diverse host-derived segments and widespread geographic links highlights interspecies transmission as a key mechanism driving the emergence of new rotavirus strains.

Of particular interest in this study are the VP2, NSP1, and NSP3 segments, whose phylogenetic analyses provide key insights into the origin, evolution, and interspecies transmission of RVA. Previous phylogenetic studies have shown that the VP2 segment of the C1 genotype in Wa-like human strains originates from pigs, suggesting that human VP2/C1 likely arose through ancient zoonotic transmission [70]. Early Wa-like human strains continue to cluster with porcine RVA [84], as also observed for the RVA/Human-wt/BRA/IAL-R406/2013/G5P[6] strain, which grouped with classical human G1P[8], G3P[8], and G4P[8] strains from the USA and Japan detected between 1974 and 1987 [97,98,99], reflecting robust genetic conservation over time. The observed similarity between human and porcine VP2/C1 strains further underscores the role of interspecies reassortment in the evolution and diversification of Wa-like human RVA.

The NSP1 segment of the RVA/Human-wt/BRA/IAL-R406/2013/G5P[6] strain belongs to the rare A8 genotype, which is uncommon in human [24,29,63,64,68,102,103], and predominantly detected in animals, particularly in swine [65,82,84,104]. Its presence in a human strain possibly reflects an interspecies reassortment event, most likely porcine-to-human transmission. Several studies worldwide have shown that human A8 NSP1 genotype cluster phylogenetically with porcine strains [24,29,63,64,102,103,104]. In the case of RVA/Human-wt/BRA/IAL-R406/2013/G5P[6], the NSP1/A8 segment clustered closely with sequences from a diarrheic piglet and an asymptomatic African green monkey from Saint Kitts, Caribbean [86]. Interestingly, Navarro et al. [86] documented interspecies transmission between porcine and monkey RVA strains.

The NSP3/T7 genotype is primarily found in animal RVA strains, particularly in pigs [65,82,86,90,91], less frequently in cattle [17,94], and occasionally in wildlife [86,100,101]. Similarly to NSP1/A8, NSP3/T7 is rare in humans and serves as a strong indicator of zoonotic transmission. In this study, the NSP3/T7 segment of RVA/Human-wt/BRA/IAL-R406/2013/G5P[6] clustered with porcine and porcine-like human strains. Comparable cases have been reported in Belgium, where a porcine-like human G9P[6] strain carried NSP3/T7 [17], and in Kenya, where a G4P[6] strain with a Wa-like backbone acquired NSP3/T7 through porcine-to-human transmission [105]. Notably, the RVA/Human-wt/BRA/IAL-R406/2013/G5P[6] NSP3/T7 segment also grouped with wildlife-derived strains from Croatian wild boars and foxes. These findings underscore the broad global distribution of the T7 genotype and highlight the contribution of sylvatic reservoirs to RVA evolution.

A key limitation of this study is the lack of patient epidemiological and vaccination data, particularly regarding potential contact with animals such as pigs, due to reliance on a passive surveillance system. This limitation restricts the ability to establish direct evidence of zoonotic transmission, as information on exposure to animal reservoirs or environmental sources is unavailable. Consequently, the interpretation of trends in diarrheal disease associated with RVA genotypes circulating in animal populations remains limited. The absence of detailed epidemiological data also hampers the differentiation between zoonotic spillover and human-to-human transmission of animal-origin strains, preventing a clearer understanding of potential exposure routes. This limitation underscores the need for integrated human–animal surveillance approaches capable of linking genomic, clinical, and ecological data within a One Health framework. Such constraints are common in studies addressing interspecies transmission, given that animal strains from the same regions where patients reside are often unavailable for comparison. Nevertheless, close human–animal interactions are well recognized as a primary driver of interspecies transmission [106].

In conclusion, the complete genome analysis of the RVA/Human-wt/BRA/IAL-R406/2013/G5P[6] strain provides valuable insights into the genetic diversity, evolution, and interspecies transmission of RVA in Brazil. The identification of a rare G5-P[6]-I1-R1-C1-M1-A8-N1-T7-E1-H1 constellation, closely related to porcine and porcine-like human strains, reinforces the role of swine as a long-standing reservoir for sporadic zoonotic infections. Moreover, the fact that G5P[6] strains do not persist in human circulation suggests that this genotype may lack the fitness required to establish sustained transmission, likely resulting in a dead-end infection. The findings underscore the importance of a One Health approach, integrating human and animal RVA surveillance to better understand cross-species transmission dynamics. Finally, the sporadic detection of G5P[6] strains in humans suggests constrained adaptability for sustained human infection, highlighting the need to monitor animal reservoirs to anticipate emerging strains of public health relevance.

## Figures and Tables

**Table 1:** Complete genotype constellations of porcine and porcine-like human RVA strains, with the genotypes of RVA/Human-wt/BRA/IAL-R406/2013/G5P[6] highlighted in blue.

Strain	Genotypes										
	VP7	VP4	VP6	VP1	VP2	VP3	NSP1	NSP2	NSP3	NSP4	NSP5
RVA/Human-wt/BRA/IAL-R406/2013/G5P[6] ^a^	G5	P[6]	I1	R1	C1	M1	A8	N1	T7	E1	H1
RVA/Human-wt/CHN/LL36755/2003/G5P[6]	G5	P[6]	I1	R1	C1	M1	A8	N1	T7	E1	H1
RVA/Human-wt/CHN/LL3354/2000/G5P[6]	G5	P[6]	I5	R1	C1	M1	A1	N1	T1	E1	H1
RVA/Human-wt/CHN/LL4260/2001/G5P[6]	G5	P[6]	I12	R1	C1	M1	A1	N1	T1	E1	H1
RVA/Human-wt/BGR/BG260/2008/G5P[6]	G5	P[6]	I1	R1	C1	M1	A8	N1	T1	E1	H1
RVA/Human-wt/ZMB/UFS-NGS-MRCDPRU4723/2014/G5P[6]	G5	P[6]	I1	R1	C1	M1	A8	N1	T1	E1	H1
RVA/Human-wt/JPN/Ryukyu-1120/2011/G5P[6]	G5	P[6]	I1	R1	C1	M1	A8	N1	T1	E1	H1
RVA/Human-wt/PRY/1809SR/2009/G4P[6]	G4	P[6]	I1	R1	C1	M1	A8	N1	T7	E1	H1
RVA/Human-wt/ARG/Arg4671/2006/G4P[6]	G4	P[6]	I1	R1	C1	M1	A8	N1	T1	E1	H1
RVA/Human-wt/ARG/Arg4605/2006/G4P[6]	G4	P[6]	I1	R1	C1	M1	A8	N1	T7	E1	H1
RVA/Human-wt/ARG/Arg12461/2014/G4P[6]	G4	P[6]	I1	R1	C1	M1	A8	N1	T7	E1	H1
RVA/Human-tc/BRA/IAL28/1992/G5P[8]	G5	P[8]	I5	R1	C1	M1	A1	N1	T1	E1	H1
RVA/Human-wt/CMR/6784/ARN/2000/G5P[7]	G5	P[7]	I5	R1	C1	M1	A1	N1	T1	E1	H1
RVA/Human-tc/GBR/ST3/1974/G4P[6]	G4	P[6]	I1	R1	C1	M1	A1	N1	T1	E1	H1
RVA/Human-tc/CHN/R479/2004/G4P[6]	G4	P[6]	I5	R1	C1	M1	A1	N1	T7	E1	H1
RVA/Human-wt/CHN/E931/2008/G4P[6]	G4	P[6]	I1	R1	C1	M1	A8	N1	T1	E1	H1
RVA/Human-wt/COD/KisB332/2008/G4P[6]	G4	P[6]	I1	R1	C1	M1	A1	N1	T7	E1	H1
RVA/Human-wt/BRA/COD379/1991/G4P[6]	G4	P[6]	I1	R1	Cx	M1	A1	N1	T1	E1	H1
RVA/Human-wt/CHN/GX54/2010/G4P[6]	G4	P[6]	I1	R1	C1	M1	A8	N1	T1	E1	H1
RVA/Human-wt/BRA/HSE005/1998/G4P[6]	G4	P[6]	I2	R2	C2	M2	A2	N2	T2	E1	H2
RVA/Human-tc/BRA/R49/1997/G1P[9]	G1	P[9]	I1	R1	C1	M2	A1	N2	T2	E1	H1
RVA/Human-wt/BEL/BE2001/2009/G9P[6]	G9	P[6]	I5	R1	C1	M1	A8	N1	T7	E1	H1
RVA/Human-tc/IND/mani-97/2006/G9P[19]	G9	P[19]	I5	R1	C1	M1	A8	N1	T1	E1	H1
RVA/Human-wt/VNM/30378/2009/G26P[19]	G26	P[19]	I5	R1	C1	M1	A8	N1	T1	E1	H1
RVA/Human-wt/BRA/rj24598/2015/G26P[19]	G26	P[19]	I5	R1	C1	M1	A8	N1	T1	E1	H1
RVA/Human-wt/PHL/TGE13-39/2013/G4P[6]	G4	P[6]	I1	R1	C1	M1	A8	N1	T1	E1	H1
RVA/Pig-wt/BEL/12R005/2012/G4P[7]	G4	P[7]	I5	R1	C1	M1	A8	N1	T7	E1	H1
RVA/Pig-tc/USA/Gottfried/1975/G4P[6]	G4	P[6]	I1	R1	C1	M1	A8	N1	T1	E1	H1
RVA/Pig-wt/BEL/12R006/2012/G3P[6]	G3	P[6]	I5	R1	C1	M1	A8	N1	T1	E1	H1
RVA/Pig-wt/JPN/BU2/2014/G5P[7]	G5	P[7]	I5	R1	C1	M1	A8	N1	T1	E1	H1
RVA/Pig-wt/BEL/12R002/2012/G5P[7]	G5	P[7]	I5	R1	C1	M1	A8	N1	T7	E1	H1
RVA/Pig-tc/USA/OSU/1975/G5P[7]	G5	P[7]	I5	R1	C1	M1	A1	N1	T1	E1	H1
RVA/Pig-wt/BRA/SUI13A/2008/G5P[13]	G5	P[13]	I5	R1	C1	Mx	A8	Nx	T7	E1	H1
RVA/Pig-wt/BRA/SUI24A/2008/G3P[13]	G3	P[13]	I5	R1	C1	M2	A8	N1	T7	E1	H1
RVA/Pig-wt/BRA/ROTA01/2013/G5P[13]	G5	P[13]	I5	R1	C1	M1	A8	N1	T7	E1	H1
RVA/Pig-wt/BRA/ROTA07/2013/G5P[13]	G5	P[13]	I5	R1	C1	M1	A8	N1	T7	E1	H1
RVA/Pig-wt/BRA/ROTA17/2013/G5P[6]	G5	P[6]	I5	R1	C1	M1	A8	N1	T7	E1	H1
RVA/Pig-wt/BRA/ROTA24/2013/G5P[6]	G5	P[6]	I5	R1	C1	M1	A8	N1	T7	E1	H1
RVA/Pig-wt/IRL/R2WTA79/2014/G5P[13]	G5	P[13]	I5	R1	C1	M1	A8	N2	T7	E9	H1
RVA/Pig-wt/ITA/3BS/2009/G9P[23]	G9	P[23]	I5	R1	C1	M1	A8	N1	T1	E1	H1
RVA/Pig-wt/THA/CMP-011-09/2009/G4P[6]	G4	P[6]	I1	R1	C1	M1	A8	N1	T1	E1	H1
RVA/Pig-tc/VEN/A131/1988/G3P[7]	G3	P[7]	I5	R1	C2	M1	A1	N1	T1	E1	H1
RVA/Pig-tc/KOR/K71/2006/G5P[7]	G5	P[7]	I5	R1	C1	M1	A1	N1	T1	E1	H1
RVA/Pig-wt/KNA/ET8B/2015/G5P[13]	G5	P[13]	I5	R1	C1	M1	A8	N1	T7	E1	H1
RVA/Pig-wt/ITA/2CR/2009/G9P[23]	G9	P[23]	I5	R1	C1	M1	A8	N1	T7	E1	H1
RVA/Wildboar-wt/HRV/DS229-Z/2020/G3P[13]	G3	P[13]	I5	R1	C1	M1	A8	N1	T7	E1	H1
RVA/Wildboar-wt/HRV/DS306-OB/2020/G3P[13]	G3	P[13]	I5	R1	C1	M1	A8	N1	T7	Ex	H1

^a^ This study strain.

## Data Availability

The nucleotide sequences determined in this study have been deposited in GenBank under the accession numbers PX448532-PX448542.
